# Analysis of strategies to attract and retain rural health workers in Cambodia, China, and Vietnam and context influencing their outcomes

**DOI:** 10.1186/s12960-018-0340-6

**Published:** 2019-01-07

**Authors:** Anna Zhu, Shenglan Tang, Nguyen Thi Hoai Thu, Leang Supheap, Xiaoyun Liu

**Affiliations:** 1 Global Health Research Center, Duke Kunshan University, Kunshan, China; 2 Duke Global Health Institute, Duke University, Durham, United States of America; 3 Department of Health Management and Organization, Hanoi Medical University, Hanoi, Vietnam; 4 Technical Bureau, National Institute of Public Health, Phnom Penh, Cambodia; 5 China Centre for Health Development Studies, Peking University, No. 38 Xueyuan Road, Haidian District, Beijing, China

**Keywords:** Contexts, Shortage and maldistribution, Attraction and retention, Rural health workers, China, Vietnam, Cambodia

## Abstract

**Background:**

Many Asia-Pacific countries are experiencing rapid changes in socio-economic and health system development. This study aims to describe the strategies supporting rural health worker attraction and retention in Cambodia, China, and Vietnam and explore the context influencing their outcomes.

**Methods:**

This paper is a policy analysis based on key informant interviews with stakeholders about a rural province of Cambodia, China, and Vietnam, coupled with a broad review of the literature.

**Results:**

Cambodia, China, and Vietnam have implemented medical education, provided financial incentives, and provided personal and professional support to attract and retain rural health workers. More socio-economic development was related to a wider range of interventions and their scope. The health system context influenced the outcomes. Increased autonomy of public hospitals attracted more health workers from rural primary health facilities in China and Vietnam. Health financing policies for universal health coverage in China and Vietnam have increased the utilization of health services. Subsidies for poor people to access health services in Cambodia have provided financial incentives to retain rural health workers. However, the dismantling of the referral system in China and Vietnam has resulted in a high rate of health workers moving from primary health facilities to higher-level hospitals while clear definition of primary healthcare package in Cambodia guided its planning of primary health workforce. The prosperous private health sector in Cambodia and Vietnam attracted more health workers from rural primary health facilities, impeded implementation and determined effectiveness of financial incentives.

**Conclusions:**

Socio-economic and health system reforms including health financing, public hospital autonomy, abolition of referral system and prosperous private sector have both positive and negative impacts on the design, implementation, and effectiveness of interventions to attract and retain rural health workers. Interventions to attract and retain health workers in rural and remote areas need to be considered within overall health system reform.

## Background

The Asia-Pacific Region has unique characteristics in terms of socio-economic and health system context. It covers both the most populous countries and small pacific island countries. Many countries have experienced a rapid economic boost in recent decades. Their health systems are undergoing significant changes in terms of health financing reform and private health sector development.

A sufficient and qualified health workforce is vital to achieving the universal health coverage as stated in the sustainable development goals (SDGs) [1]. However, many countries suffer from a shortage and maldistribution of human resources for health (HRH), especially in Asia-Pacific countries [2–4]. In 2012, there were 1.4 health workers per 1000 people in Cambodia [4], which fell below the critical shortage threshold defined by the World Health Organization (WHO) as 2.28 doctors, nurses, and midwives per 1000 people [5, 6]. Approximately 40% of general physicians and 74% of specialist physicians were concentrated in the capital city Phnom Penh [4]. In China, the density of health workers in the urban areas was 10.2 per 1000 people while it was only 3.9 in the rural areas in 2015 [7]. In Vietnam, health workers per 1000 people in 2012 was 2.6. About 82% of pharmacists, 59% of doctors, and 55% of nurses provided health services for only 30% of the total population in the urban areas [3].

WHO in 2010 recommended key interventions to attract and retain health workers in rural areas [8]. These interventions included medical education, compulsory regulation, financial incentives, and personal and professional support. However, it is well recognized that there is no single intervention suitable in all countries [9]. Interventions to strengthen the rural health workforce need to be tailored according to the specific socio-economic and health system context of individual countries [10]. Moore and colleagues pointed out that implementation was often localized and diversified due to existing contexts, which in turn led to variations in effectiveness [10]. Understanding how interventions work in different contexts can also facilitate effective evaluation and scale up to other settings [11]. Nevertheless, there was little evidence on the implications of contexts on rural health workforce strengthening. The potential impact of different individual contexts are often neglected when interventions are designed and implemented, resulting in negative consequences, such as low cost-effectiveness, poor health service performance, and ultimately poor health for the population [12]. Liu and colleagues summarized the impact of political, economic, and social factors and health system and implementation process-related contexts on compulsory and incentive strategies [13].

This study aims to describe the strategies supporting rural health worker attraction and retention in Cambodia, China, and Vietnam and explore the context influencing their outcomes. Through presenting the tri-country case studies, this study also summarizes the key lessons for the policy makers and other countries with similar contexts to strengthen and rebalance their health workforce.

## Methods

This study is a policy analysis built around key informant interviews with stakeholders about a rural province of Cambodia, China, and Vietnam. It explores how different contexts influence HRH strengthening in Cambodia, China, and Vietnam. Socio-economic development status was one of the key considerations in selecting these countries. Cambodia, China, and Vietnam are upper-middle-income, middle-income, and low-income countries, respectively. They represent different HRH situations. The authors had existing collaborations with country partners.

In each country, one rural province was selected as the study site (Guangxi in China, Bac Giang in Vietnam, and Kampong Chhnang in Cambodia). The provinces were recommended through known collaborations of the authorship team based on the follow selection criteria: (1) rural and remote location, (2) with interventions on attraction and retention of rural health workers, (3) willingness to collaborate. Some key informants were also recruited from the capital cities.

In each country, four categories of key informants, including national policy makers, academic experts, local health managers, and rural health workers, were recruited using a purposive sampling strategy so that they could provide broad and rich information on the contextual factors and their implications on health workforce attraction and retention. The interviewees were recommended and recruited through the existing local collaborations.

In-depth interviews were conducted in 2016. The interviews covered four themes, including overall HRH situation, interventions to attract and retain rural health workers, implementation and effectiveness of such interventions, and each country’s context. Each interview lasted approximately 1 h and was conducted in a quiet meeting room. Informed consent was obtained from all interviewees, and interviews were tape recorded, with one exception as the interviewee declined to be recorded, in this instance written notes were used to record the interview. Additionally, national policy documents, country study reports, and gray literature recommended by the key informants were also collected.

Local collaborators acted as interpreters when interviewees could only speak the local language (Vietnamese and Khmer). In total, 28 key informants from Cambodia, China, and Vietnam were interviewed (Table 1).

**Table 1:** Summary of key informants in Cambodia, China, and Vietnam

Country	National policy makers	Academic experts	Health managers	Rural health workers	In total
Cambodia	2	2	4	1	9
China	1	1	3	5	10
Vietnam	1	2	3	3	9
Total	4	5	10	9	28

Data was analyzed using a thematic framework approach with developing both deductive and inductive codes [14, 15]. All interview recordings were transcribed verbatim into English. First, the thematic framework was developed using a deductive approach based on existing theories and frameworks as reflected in the interview topic guides [14, 16]. Inductive codes were added when new themes emerged from the interview transcripts [14]. The transcripts were then coded to test, validate, and refine the themes. Information under each theme was retrieved and summarized to present the emerging findings [16]. Key interventions from three countries were summarized separately. The contexts were divided into two categories—socio-economic and health system contexts. The socio-economic category included economic development and social development. The health system category included decentralization, health financing, primary service delivery system, and private health sector. Emphasis was put on the implications of these contexts on the design, implementation, and effectiveness of interventions.

## Results

### Human resource strategies employed

#### Cambodia

Medical education, regulation, and personal and professional support were the key interventions reported in Cambodia.

Interviewees explained that due to a weak health workforce and a high maternal mortality rate in rural areas, the Cambodian government emphasized medical education of mid-level health workers (midwives and nurses). A 1-year medical education program for primary nurses and midwives and a 3-year program for secondary nurses and midwives were implemented in 1990s. Students with 12-year primary education were eligible to receive free medical education at the regional training centers in four provinces, including Stung Treng, Kampot, Kampong Cham, and Tbong Khmum. In terms of regulation interventions, Ministry of Health and Provincial Health Department worked together to allocate the rural health workers based on examination performance. In terms of personal and professional support, the Cambodian government announced in 2016 that all health workers (in urban and rural areas) should be promoted to civil servants which implied permanent jobs and better social welfare benefits.

#### China

The key informants explained that China implemented a comprehensive package of interventions, including medical education, financial incentives, regulation, and personal and professional support to rural health workers.

Special medical education programs were introduced and implemented in China to equip rural primary health facilities with more qualified health workers. Central government issued a medical rural bonded scholarship in 2010, to train physicians with bachelor degrees to work in rural township health centers in all central and western provinces. Eligible candidates received 5 years of free medical education and a monthly stipend in return for 6 years of compulsory service in rural areas. This program has recruited 5000 medical students every year since 2010 [17]. A cohort study showed that 90.7% of medical graduates from this program adhered to the contract and worked in rural health centers after graduation [18]. Additionally, the Ministry of Education initiated a new medical education program in 2010 to train assistant physicians for rural clinics. Graduates received an associate degree after a 3-year education and were limited to practice at rural health facilities [19]. One-year compulsory rural service before promotion was announced to support rural health facilities in several provinces, like Hubei, Hebei, and Shaanxi Province.

China also employed interventions using financial incentives to attract and retain rural health workers. In 2008, the central government founded a financial incentive program that subsidized the annual income of physicians by 20 000 RMB (about 2914 USD) in rural areas of eight pilot provinces. This program was later scaled up to 23 central and western provinces. According to one key informant who was responsible for the program evaluation, the program successfully recruited 1080 general physicians for 828 township health centers in 218 rural counties.

After 2009, the government improved personal and professional support to strengthen capacity of primary health facilities in both urban and rural areas. After the reform, the government is responsible for financing primary health facilities, including investment in infrastructure construction, equipment procurement, and human resources. These measures largely improved rural working conditions [2]. In addition, between 2009 and 2013, the government allocated special funding to subsidize various in-service training for rural health workers [2]. Furthermore, partnerships were developed between urban and rural health facilities. The former provided long-term technical assistance for the latter, which helped mitigate professional isolation in the rural areas [2].

#### Vietnam

The Vietnamese government applied in-service training, financial incentives, and personal and professional support to build a strong rural health workforce.

Key informants stated that in-service training was widely used to strengthen the capacity of rural health workers although it did not directly expand the pool of rural health workers. In 2012, 1816 training projects were conducted, mostly in the rural areas. Some of the projects were long-term training such as upgrading the qualifications of assistant physicians.

From 2009 to 2014, the Vietnamese government issued five decrees and circulations including financial incentive terms to improve income among rural health workers. For instance, under Decree No. 64, rural health workers became eligible for an additional 70% of basic salary as allowance for first 5-year rural services. Most financial incentives were modest and only available for a few years. In addition, some local governments in developed regions also provided higher income and housing subsidies to attract rural health workers.

In terms of personal and professional support, key informants informed that the central government announced Decree No. 117 in 2014 which promoted health workers with more than 3 years of rural service to civil servants. A civil servant position was regarded as a stable and promising career and therefore provided strong incentive.

### Context influencing strategies and their outcomes

Combining information from literature review and key informant interviews, Table 2 illustrates the positive or negative impact the various socio-economic and health system contexts had on the design, implementation, and effectiveness of interventions.

**Table 2:** Overview of socio-economic and health system contexts in Cambodia, China, and Vietnam

Category	Contexts	Description	Implications	Number of coded segments
Socio-economic contexts	Economic development	There are economic disparities across and within the countries of Cambodia, China and Vietnam.	The density of health workers is positively associated with the size of economies across and within the countries.	10
	Social development	Cambodia has poor social development.	In Cambodia, poor primary education in the rural areas hampers the recruitment of candidates for medical education.	7
Health system contexts	Decentralization	China and Vietnam increased autonomy of public hospitals. Cambodia decentralized the operation of health centers.	Increased autonomy of public hospitals in China and Vietnam resulted in brain drain at rural primary health facilities. In Cambodia, decentralization allocated more funding to the health centers.	15
	Health financing	China established New Rural Cooperative Medical Scheme. Vietnam conducted mandatory health insurance. Cambodia applied health equity fund, and official development assistance as financial incentives.	In China and Vietnam, health insurance schemes for universal health coverage improved demand for and utilization of health services in the rural areas. In Cambodia, pro-poor health financing policies motivated rural health workers with more financial incentives.	12
	Primary service delivery system	Vietnam and China abolished referral system. Cambodia applied Minimum Package of Activities.	In Vietnam and China, abolition of referral system led to brain drain at primary health facilities. In Cambodia, implementation of MPA partly caused no physicians at primary health facilities.	10
	Private health sector	All three countries had prosperous private health sector.	In Vietnam and Cambodia, prosperous private health sector led to brain drain at rural primary health facilities, impeded implementation and determined effectiveness of financial incentives.	6

### Socio-economic contexts

The size and distribution of health workers was largely determined by economic development status. There was a positive relationship between GDP per capita and density of health workers. In 2016, GDP per capita was 8123 USD in China, which was much higher than in Vietnam (2171 USD) and Cambodia (1270 USD) [20]. Meanwhile, the number of health workers per 1000 population in China (4.9 in 2012) was also larger than in Vietnam (2.6) and Cambodia (1.4) [2–4].

Economic disparities were also reflected in the design of interventions. According to the interviews, China had the financial capacity to scale up financial incentives for general physicians who agreed to work in rural areas. With a weaker economy, Cambodia chose instead to emphasize the primary and associate medical education on midwives and nurses.

Other social development factors also influenced the design of interventions. In Cambodia, most students dropped out of schools by the 8th or 9th grade. This made recruiting candidates that met the 12-year primary education requirement to enroll in midwife and nursing training programs difficult. In order to recruit enough candidates, the government was forced to reduce the education requirement to the 7th grade level.

We have lack of people with enough educational background, like the students with high school diploma…The civil servant standard requires the students to have 12-year primary education plus another 1-year medical education to become primary midwives and nurses, and 12 plus 3 to become associate (midwives and nurses). Most students in the communities have only 7-year schooling… That’s why we don’t have many health workers. (National policy maker from Cambodia)

### Health system contexts

Health system contexts included decentralization, health financing, primary service delivery, and private health sector. They had varied implications on the implementation and effectiveness of interventions.

#### Decentralization

Decentralization in different forms was widely reported, as one of the main drivers of a stronger rural health workforce. In China and Vietnam, the increased autonomy of public hospitals (as one form of decentralization) resulted in loss of health workers from primary health facilities. Key informants from Vietnam reported that government Decree No.43 issued in 2006 was to grant more autonomy to public hospitals on health personal and financial management. As a result, public hospitals were encouraged to recruit more qualified health workers to produce higher revenues through increasing health service supply. Qualified health workers became concentrated in urban public hospitals while leaving the positions vacant at rural primary health facilities.

Decree 43, was issued about 10 years ago…if any health facility has enough capacity that they can run by themselves in terms of financing, and they can provide the salary for their health workers, they can recruit…Hospital leader has been granted more autonomy to create the revenue of hospital by providing more health services and higher level of additional income, which is enough to attract health workers to work in our province. (Health managers from Vietnam)

A similar situation occurred in China. In the early 1990s, the government dramatically decreased financial subsidies to public hospitals and granted them with more financing autonomy. Public hospitals started to invest more on infrastructure, equipment, and health workforce to expand health service supply in pursuit of profit from increased medical tests, drug sales, and other services. This situation left primary health facilities (especially in rural areas) vulnerable to losing their qualified health workers to better paying positions in public hospitals [21].

Key informants from Cambodia explained that decentralization was implemented to strengthen rural health workforce development. Health centers were encouraged to develop their own annual development plan and received performance-based funding from the local government. This policy increased direct funding to health centers for them to recruit more health workers.

Decentralization is managed by Ministry of Interior…The process is rolling out and requires the health staff in health centers to be actively involved to develop their annual action plan. Their long-term funding will depend on their performance…The amount of money going down is also increasing quite substantially… If this provides better and sustainable funding in the health center in the long run, it will help retain the health staff. (Academic expert from Cambodia)

#### Health financing

Health financing policies in all three countries gave rural health facilities more leverage to attract and retain health workers. Both Chinese and Vietnamese governments increased financial investment to expand health insurance coverage. In China, the New Rural Cooperative Medical Scheme established in 2003 covered the majority of rural population [2]. Under Revised Health Insurance Law, Vietnam implemented compulsory health insurance in 2015 which covered about 81% of the population by 2016 [22]. Improved health coverage greatly increased the demand for health services, especially in the rural areas [2]. The increasing utilization of rural health services led to increasing demand for qualified health workers, which in turn prompted the need to scale up interventions for HRH attraction and retention.

In Cambodia, key informants explained that health financing policies provided extra financial incentives for rural health workers. First, a health equity fund was established in 2000 to subsidize poor populations’ access to health services and was also used to provide financial incentives for rural health workers [23]. Second, official development assistance (ODA) provided another form of financial resources to increase pay for rural health workers. ODA contributed up to 20% of total health expenditure in 2012 [4]. ODA-funded disease prevention and health promotion programs provided economic benefits to rural health workers through implementing activities such as data collection and patient referrals.

Health equity fund would be quite significant because it can be used by health staff. They provide quite substantial supplement to the income in particular poor areas…Global Fund and JICA are still paying incentives. They have directed more funding on the staff working in the rural facilities…village malaria workers receive regular payment because they do quite a lot of important work in terms of quick test for malaria and also data collection. (Academic expert from Cambodia)

#### Primary service delivery system

The Vietnamese government abolished its referral system in January 2016. Key informants reported that this reform drove more patients to visit district hospitals and significantly decreased the patient volume in health centers. Reduced outpatient services decreased service revenues and available financial incentives for rural health workers; as a result, health workers fled away from rural primary health facilities. The same situation occurred in China in the 1980s when the gate keeping system was abolished and patients were given freedom to choose public hospitals as their first contact [24].

In the past, every insured patient has to go to the health centers first while now they can directly go to the district hospitals or other health centers if they like…Patients have tendency to go to the district hospitals because there are more health services they can use there. The patients think that health workers at district hospitals have higher qualification and are better equipped. The list of essential medicine is also more diverse in the district hospitals. (Rural health workers from Vietnam)

In Cambodia, the government issued Minimum Package of Activities (MPA) in 1996 as part of the operation guidelines for health centers, which included detailed requirements on the service provision, health workforce management, and development for health facilities. Defined by MPA, the primary and secondary midwives and nurses were qualified and responsible for health services at primary health centers. If advanced health services were needed, patients could visit the physicians at district- or high-level hospitals. Key informants argued that with current MPA, only nurses and midwives, rather than physicians, were needed at primary health centers to provide relevant services. MPA helped ensure that the existing health workforce was efficiently utilized.

It relates to our Minimum Package of Activities and Comprehensive Package of Activities. Health centers only provide health education, obstetric activities and immunization. When the health services are not available in the health centers, they are referred to the hospitals where they have doctors. In the future, if Minimum Package of Activities and Comprehensive Package of Activities changed, we also change in accordance… If the population size increases, they have to upgrade their services. The health centers will become hospitals, which means that they need doctors. If the services don’t need to be there, we don’t want to use our staff in low efficiency. (National policy maker from Cambodia)

#### Private health sector

In Vietnam, the private health sector provided 60 to 75% of ambulatory health services, 40% of outpatient services, and 4% of inpatient services [3]. Over half of the public health workers also had private practice. In Cambodia, about one quarter of overall health workers were private healthcare providers. Fifty-six percent of public health workers had dual practice in the private health sector [25]. Dual practice had significant contribution to health workers’ income. Their monthly income increased from 169 to 400 USD for nurses and midwives and from 320 to 1 500 USD for specialist physicians [25].

According to the key informants, the booming private health sector in Vietnam and Cambodia became a threat to the implementation of financial incentives to attract and retain rural health workers. Most private hospitals were established in urban cities, and they offered higher salaries to recruit staff. Limited opportunities for private practice in the rural areas made the rural positions less attractive.

If there is no opportunity for staff to practice in the rural areas outside the public roles, it is very hard to retain the staff… The population was small and the opportunity for doing private practice is very limited. So the specialist staff don’t stay there and they go to province with large population. (Academic expert from Cambodia)

In the past decade, the private health sector in China has also dramatically increased. Proportion of private hospitals increased from 10% in 2002 to 53% in 2015 [2]. However, the size of private hospitals was usually small, and they only contributed to 12% of outpatient services in 2015 [2]. Key informants reported that public hospitals still played a dominant role in China and could provide better career development opportunities. Private hospitals were reported to experience difficulties in recruiting qualified physicians although they often offered higher compensation. Few physicians from public hospitals had dual practice in the private health sector.

## Discussion

Cambodia, China, and Vietnam have made substantial efforts to attract and retain rural health professionals by implementing a wide range of interventions recommended by WHO, including medical education, financial incentives, regulation, and personal and professional support [8].

Socio-economic development may have critical impacts on the design, implementation, and effectiveness of interventions. Disparities in socio-economic development status among countries will largely impact the design of interventions to attract rural health workers. Countries with increasing financial resources (like China) can better afford interventions to strengthen rural HRH. Mandatory rural bonded scholarships are much more likely to influence rural retention in countries where conformity to prescribed behavior is strong (Japan) and legal contracts are enforced (Australia) [26, 27].

Social culture may have specific effects on program development and implementation. Seniority is the primary criteria for professional promotion in many Asia-Pacific countries. Under this context, performance-based incentives are hard to scale up and the effects are limited, especially for the younger health workers. Some other Asian cultures value strong social accountability to serve rural areas. In the Philippines, it was reported that social accountability motivated medical graduates to work for the local health facilities [28].

Health systems were an important contextual factor for the development of HRH. Pro-poor health financing policies for universal health coverage increased the demand for, and utilization of, health services and provided financial incentives to motivate rural health workers, like health equity fund in Laos and Cambodia [29]. However, the increased financial incentives may also be compromised by the increasing workload which may result in a high turnover rate of primary health workers as the case in Thailand [30]. The blossoming private health sector in the Asia-Pacific Region can negatively impact the effectiveness of interventions within and across countries, given that the attractiveness of working in the private sector outweighed the financial and non-financial incentives offered by the government in rural primary health facilities [31].

Regardless of the importance of intervention design and implementation, contextual factors are often overlooked when making domestic and global policy recommendations. Although some studies and key informants in our case studies stressed the importance of contextual factors, they were seldom analyzed in a systematic way during the policy formulation and implementation process [13].

This policy narrative highlights the fact that using interventions to strengthen HRH as an individual health system building block in isolation may not result in attracting and retaining health workers in rural and remote areas. Health systems have many interacting parts that operate within social, economic, and political contexts. Expecting health workers to stay in facilities that have inadequate supplies and equipment, or where living conditions and educational facilities deter family posting, is neither realistic nor practical—even if the staff concerned receive special training and financial incentives. The effectiveness of interventions is inevitably influenced by the effectiveness of governance (in the health sector and beyond) and the relationship between the public and private services. This is especially the case in those countries with limited financial resources, poor governance, limited administrative and managerial capacity, and high levels of social, geographical, and gender inequity. In these circumstances, shortage of well-qualified health personnel in rural areas might be inevitable. Single, poorly funded interventions with little political support could hardly be successful.

### Limitations

The evidence in this study was mainly qualitative and could only discuss opinions of stakeholders and written material rather than actually testing outcomes. A limited number of stakeholders was interviewed to exploring this topic in rural provinces of three countries, but it still provides some useful insights. More in-depth case studies would be needed to explore particular human resource questions relative to context.

## Conclusions and recommendations

Cambodia, China, and Vietnam implemented various interventions to strengthen their rural health workforce, including medical education, financial incentives, and professional support. Socio-economic development has impact on the size of the health workforce and the range and scope of interventions. Pro-poor health financing systems and clear definition of primary health care package have positive impacts, while increasing management autonomy of public hospitals, abolition of referral systems, and the growth of prosperous private health sectors have negative impacts on the design, implementation, and effectiveness of interventions to attract and retain rural health workers.

Landscape analysis on context factors should be conducted when designing, implementing, and evaluating interventions. Interventions to attract and retain health workers in rural and remote areas should not be implemented in isolation. Rather, they should be integrated into overall health system reform, which includes reforms that improve working conditions and governance. When developing a health system reform, the potential impacts on the implementation and effectiveness of interventions to attract rural health workers should be systematically monitored in order to achieve universal health coverage and the sustainable development goals.

## Abbreviations

HRH: Human resources for health
MPA: Minimum Package of Activities
ODA: Official development assistance
SDG: Sustainable development goal
WHO: World Health Organization
